# Editorial: Impact of diet-related disorders on musculoskeletal health

**DOI:** 10.3389/fnut.2023.1301837

**Published:** 2023-10-09

**Authors:** Caterina Conte, Paola De Luca

**Affiliations:** ^1^Department of Human Sciences and Promotion of the Quality of Life, San Raffaele Roma Open University, Rome, Italy; ^2^Department of Endocrinology, Nutrition and Metabolic Diseases, Istituto di Ricovero e Cura a Carattere Scientifico (IRCCS) MultiMedica, Sesto San Giovanni, Italy; ^3^IRCCS Istituto Ortopedico Galeazzi, Laboratorio di Biotecnologie Applicate all'Ortopedia, Milan, Italy

**Keywords:** nutrition, metabolism, musculoskeletal system, sarcopenia, osteoarthritis, inflammation

In an era where fast food and sedentary lifestyles have become the norm for many people, the interplay between nutrition, metabolic health, and musculoskeletal wellbeing is a topic of increasing importance (1–3). The impact of diet-related disorders on musculoskeletal health has been underestimated and often overlooked. However, as our knowledge deepens, the connection between nutrition and musculoskeletal health becomes glaringly apparent. This editorial aims to shed light on this connection, drawing from a collection of articles that explored the profound implications of the impact of diet-related disorders on musculoskeletal health.

The first study, by Zhang et al. delved into the complex relationship between socioeconomic status (SES), body mass index (BMI), and bone mineral density (BMD). Their research revealed that SES has a significant influence on BMD, which is mediated in part by BMI. Socioeconomic disparities can affect the integrity of bones, and this finding underscores the importance of addressing not only nutritional habits but also the broader social determinants of health. This research emphasizes that addressing disparities in bone health requires a multifaceted approach that includes not only dietary modifications but also socioeconomic interventions.

The second study, by Herrero-Manley et al. highlighted the role of serum lipid and inflammatory biomarkers in the onset and progression of early knee osteoarthritis. This study provides compelling evidence that higher total cholesterol, LDL, uric acid, and CRP levels are associated with increased pain intensity, disability, and decreased functional performance. It is evident that dietary habits and their effects on lipid profiles can play a significant role in the development of musculoskeletal disorders, particularly osteoarthritis.

Amirkhizi et al. investigated the association between vitamin D status, oxidative stress, and matrix metalloproteinases (MMPs) in patients with knee osteoarthritis. Their study revealed that vitamin D deficiency was linked to increased oxidative stress and MMP activity, both of which contribute to the progression of this painful condition. This study highlights the crucial role of maintaining an adequate vitamin D status, which can be influenced by diet and sunlight exposure, in preventing oxidative processes that can damage joints.

In a fourth study, Cui et al. explored the association between digestive diseases and sarcopenia. Their study underscores the intriguing association between these conditions. Their findings indicate that individuals with digestive diseases have a higher risk of developing sarcopenia, a condition characterized by reduced muscle mass and function. This research not only raises questions about the underlying mechanisms but also emphasizes the potential of nutritional interventions to prevent or mitigate these health issues.

As we peruse these four studies, a common thread emerges: nutrition and its effects on metabolic health are intimately intertwined with the wellbeing of our musculoskeletal system. This Research Topic aimed to globalize the work of scientists studying how nutrition and metabolism affect the musculoskeletal system, and these articles collectively bring us closer to that goal.

The implications of this research are substantial. This calls for a re-evaluation of nutritional interventions and an increased awareness of the detrimental impact of diet-related disorders on musculoskeletal health, particularly with aging. Patient-tailored dietary interventions can not only help prevent these disorders but might also play a role in the management and treatment of musculoskeletal diseases (4, 5).

Although these findings offer valuable insights, they also prompt new questions. How can this knowledge be translated into practical dietary recommendations? How can we make these findings accessible and understandable to the public? Answering these questions will require multidisciplinary collaboration involving healthcare professionals, policymakers, and the public.

In conclusion, the relationship between nutrition and musculoskeletal health is far more profound than previously thought. The articles featured in this Research Topic laid a strong foundation for future investigations and policy development. It is imperative to acknowledge the role of nutrition in preserving and enhancing musculoskeletal health, promoting a balanced and wholesome diet, and continuing to explore targeted dietary interventions.

## Author contributions

CC: Writing—review & editing. PD: Writing—review & editing.
